# Gestational Diabetes Associated with Postpartum NAFLD Risk Meta-Analysis: Evidence for Sustained Metabolic Dysfunction Beyond Pregnancy

**DOI:** 10.3390/jcm15031209

**Published:** 2026-02-04

**Authors:** Milica Stoiljkovic, Katarina Lalic, Tanja Milicic, Ljiljana Lukic, Marija Macesic, Jelena Stanarcic Gajovic, Mina Milovancevic, Sara Cvijanovic, Djurdja Rafailovic, Stefan Maric, Milica Vujasevic, Nina Krako Jakovljevic, Kasja Pavlovic, Miroslava Gojnic, Nebojsa Lalic, Aleksandra Jotic

**Affiliations:** 1 Clinic for Endocrinology, Diabetes and Metabolic Diseases, University Clinical Center of Serbia, Doktora Subotica 13, 11000 Belgrade, Serbia; katarina.s.lalic@gmail.com (K.L.); icataca@gmail.com (T.M.); ljikson17@gmail.com (L.L.); macesicmarija@gmail.com (M.M.); stanarcicjelena@gmail.com (J.S.G.); milovancevicmina3@gmail.com (M.M.); djurdja.rafailovic@gmail.com (D.R.); ninakrako@gmail.com (N.K.J.); kasja.pavlovic@med.bg.ac.rs (K.P.); aleksandra.z.jotic@gmail.com (A.J.); 2 Faculty of Medicine, University of Belgrade, Doktora Subotica 8, 11000 Belgrade, Serbia; cvijanovic.sara99@gmail.com (S.C.); vujasevicmilica98@gmail.com (M.V.); miroslavagojnicdugalic@yahoo.com (M.G.); nebojsa.m.lalic@gmail.com (N.L.); 3 Clinic for Gynecology and Obstetrics, University Clinical Centre of Serbia, Visegradska 26, 11000 Beograd, Serbia; 4 Department of Medical Sciences, Serbian Academy of Sciences and Arts, Kneza Mihaila 35, 11000 Belgrade, Serbia

**Keywords:** gestational diabetes, non-alcoholic fatty liver disease, long-term risk metabolic dysfunction, meta-analysis

## Abstract

**Background/Objectives:** Gestational diabetes (GD) is a well known risk factor for future metabolic diseases. However, the long-term time-dependent risk of non-alcoholic fatty liver disease (NAFLD) remains unexplored. The aim of this meta-analysis was to quantify the long-term risk of NAFLD in women with previous GD and evaluate if the risk persists beyond the postpartum period. **Methods:** A systematic search was performed in PubMed using appropriate medical subject headings to identify trials evaluating the incidence of NAFLD among women with previous GD compared to those with normal glucose tolerance (NGT). Studies reporting adjusted risk estimates with a follow-up duration beyond pregnancy were included. Data were extracted and analyzed using relevant statistical methods, with the level of significance at *p* < 0.05. **Results:** A total of four studies (N = 2873) were included in the meta-analysis. Women with previous GD had a 2.46-fold higher odds of NAFLD compared to those with NGT (95% CI 1.88–3.23, *p* < 0.001). The slope for years since delivery was not significant (β = 0.001 per year, 95% CI −0.037 to 0.040, *p* = 0.901), indicating that the likelihood of NAFLD in women with prior GD did not change over time. **Conclusions:** GD is associated with a substantially increased and sustained risk of NAFLD, persisting decades beyond pregnancy. These findings identified GD as a potential early risk marker of future liver outcomes and highlight the need for long-term metabolic screening and preventive strategies for this high-risk population.

## 1. Introduction

Metabolically dysfunction-associated steatotic liver disease (MASLD), previously termed non-alcoholic fatty liver disease (NAFLD), has become the most common chronic liver disease. Unlike NAFLD, the definition of MASLD requires at least one more cardiometabolic risk factor, which better reflects its underlying pathophysiology and clinical outcomes [1]. Prior studies indicate a rising prevalence of MASLD, affecting over 30% of the general population [2], with the greatest increase observed under 40 years of age [3]. MASLD comprises a spectrum of conditions ranging from simple steatosis to cirrhosis, and MASLD-related cirrhosis has emerged as the leading indication for liver transplantation in women [1,4]. The presence of MASLD is strongly associated with type 2 diabetes (T2D), obesity, and other cardiometabolic risk factors [1,5]. Although gestational diabetes (GD) is defined as hyperglycemia first recognized during pregnancy, it is followed by an increased long-term risk of cardiometabolic disease later in life [6,7].

Both MASLD and GD share overlapping pathophysiological mechanisms involving metabolic dysregulation. Insulin resistance plays a pivotal role in both disorders, disrupting glucose and lipid homeostasis and contributing to hepatic fat accumulation. Moreover, both conditions are characterized by disturbances in adipokine profiles, including decreased adiponectin and increased leptin, which further exacerbate systemic inflammation. Emerging evidence also suggests mitochondrial dysfunction in both GD and MASLD, promoting cellular injury and metabolic inflexibility. The unfavorable metabolic milieu that persists postpartum among women with prior GD, as outlined above, predisposes them to an increased risk of MASLD [8,9,10,11,12]. These shared molecular disturbances imply a potential mechanistic continuum between GD and subsequent MASLD development, particularly during the postpartum period. However, the trajectory and time course of postpartum MASLD risk remain underexplored.

Several studies have evaluated the association between GD and subsequent MASLD, but the findings remain inconsistent, likely due to differences in sample size, diagnostic criteria, and duration of follow-up. In that context, it remains unclear whether the risk of MASLD in women with GD emerges only during the early postpartum period or persists throughout the lifespan. These gaps highlight the need for a comprehensive synthesis of existing evidence to better define how MASLD risk evolves after GD.

To address this, we conducted a meta-analysis to assess and quantify not only the risk of NAFLD in women with prior GD but also to explore whether this risk varies with the duration of follow-up.

## 2. Materials and Methods

This meta-analysis was conducted in accordance with PRISMA 2020 guidelines. Analyses followed predefined objectives that are transparently documented herein. The review protocol was not prospectively registered (e.g., in PROSPERO); however, the research question, eligibility criteria, and analytical strategy were defined prior to data extraction.

### 2.1. Eligibility Criteria

Eligible studies were observational (cohort, case–control, or cross-sectional) and evaluated a history of GD as the primary exposure, confirmed through clinical or registry data, with a comparator group of women without GD. Outcomes included non-alcoholic fatty liver disease (NAFLD) or metabolic dysfunction-associated steatotic liver disease (MASLD) diagnosed by imaging (ultrasound, CT, MRI, FibroScan/CAP) or validated index, the Fatty Liver Index (FLI) [1,13]. We prioritized the LFS (validated against H-MRS/MRI-PDFF) because MR-based fat quantification is more accurate and sensitive, particularly for mild steatosis, than ultrasound, which is qualitative [14,15], and FLI and HSI were validated against ultrasound data [16,17]. Studies had to provide, or allow calculation of, effect sizes (odds ratios, hazard ratios, or β coefficients with 95% confidence intervals) [18].

None of the included studies assessed hepatic steatosis during pregnancy; therefore, baseline NAFLD/MASLD status in gestation was unknown, and incident postpartum NAFLD cannot be distinguished from pre-existing disease.

Exclusion criteria included GD exposure identified only by unvalidated self-report, studies conducted during pregnancy (e.g., acute fatty liver of pregnancy), reviews and Mendelian randomization analyses, animal experiments, and studies where GD was not the direct exposure of interest (e.g., breastfeeding, contraception, or pharmacological interventions in prior GD populations).

### 2.2. Information Sources and Search Strategy

We systematically searched PubMed from inception to 30 June 2025, using a combination of medical subject headings terms and free-text keywords related to GD and NAFLD. The search was limited to PubMed as the primary biomedical database and was supplemented by manual screening of reference lists from included articles and relevant review papers to identify additional eligible studies. Furthermore, the search was limited to human studies conducted in adult women and published in English. Reference lists of included studies and relevant reviews were also screened manually.

### 2.3. Study Selection

Four reviewers independently screened titles and abstracts, followed by a full-text review of potentially eligible articles. Discrepancies were resolved by discussion. The search yielded 70 records; a total of 55 were excluded at screening, and 15 full texts were assessed. Of these, 11 were excluded, leaving 4 studies for quantitative synthesis. Study selection is summarized in the PRISMA flow diagram (Supplementary Materials Figure S1).

### 2.4. Included Studies and GD Diagnostic Criteria

Four studies were retained: Ajmera et al. (2016, USA, CT-based NAFLD diagnosis), Donnelly et al. (2019, Denmark, fatty liver indices derived from registry and biochemical data 9–16 years postpartum), Forbes et al. (2011, Europe, ultrasound prevalence), and Kubihal et al. (2021, India, ultrasound and FibroScan CAP) [19,20,21,22]. As all the included studies predated the MASLD terminology, we retained the original NAFLD definition to ensure comparability across studies. GD ascertainment was based on standardized criteria reflecting evolving practice. Ajmera et al. used validated self-report confirmed against medical records, Donnelly et al. used national registry data with a 2 h 75 g OGTT, Forbes et al. applied the WHO 1999 criteria, and Kubihal et al. used the IADPSG 2010 thresholds.

### 2.5. Data Extraction and Risk of Bias

Data were extracted independently by two reviewers into a standardized template, including study design, sample size, population characteristics, exposure and outcome ascertainment, and effect estimates. Adjusted estimates were preferentially recorded. The risk of bias was assessed using the Newcastle–Ottawa Scale (NOS) across the selection, comparability, and outcome domains; studies scoring ≥ 7 were considered of high quality. Validity of NAFLD ascertainment by imaging versus indices was also noted.

### 2.6. Effect Measures and Synthesis

Effect sizes were transformed to log odds ratios and pooled using a random-effects model with restricted maximum likelihood (REML) estimation, weighted by inverse variance. Results were expressed as odds ratios with 95% confidence intervals (CIs). Between-study heterogeneity was evaluated using Cochran’s Q statistic, τ^2^, and I^2^. Sensitivity analysis was performed using a leave-one-out approach, whereby each study was sequentially excluded to assess the influence of individual studies on the pooled effect estimate.

A random-effects meta-regression (REML) was performed to examine whether follow-up duration modified the association between prior GD and subsequent NAFLD risk. Follow-up duration was defined as the interval between delivery of the index pregnancy and the assessment of NAFLD. When studies reported group-specific or median recall times, a single study-level value was calculated as the sample-size-weighted mean or the reported median. Follow-up years were modeled as a continuous covariate. Models estimated the change in odds ratio per 5-year increase in follow-up (OR5y = eβ × 5). The Knapp–Hartung adjustment was applied for standard errors, and R^2^ statistics quantified the proportion of between-study variance explained by the moderator.

### 2.7. Additional Analyses

Given the small number of included studies (N = 4), formal statistical tests for publication bias (e.g., funnel plot asymmetry or Egger’s regression) were not considered reliable and were therefore not used for inference. Instead, potential small-study effects were explored qualitatively by examining the consistency of effect estimates, study size, methodological characteristics, and the stability of pooled results in leave-one-out sensitivity analyses.

All analyses were performed in SPSS Statistics 31.0 (Meta-Analysis module), with a two-tailed significance threshold of *p* < 0.05.

### 2.8. Ethical Consideration

Ethical approval was not required for this meta-analysis, as the study involved the analysis of data that was already published. All included studies had obtained ethical approval from relevant institutional review boards, as stated in the respective publications. No unpublished or individual patient data were used in this analysis.

### 2.9. Supplementary Materials

The full PubMed search strategy, PRISMA 2020 checklist, PRISMA 2020 flow diagram, leave-one-out analysis, risk-of-bias table, and detailed data extraction sheet are provided in Appendix A.

## 3. Results

### 3.1. Study Characteristics

Four observational cohort studies were included in the quantitative synthesis, with follow-up periods ranging from 1.33 to 25.00 years. All studies compared women with a history of gestational diabetes (GD) with controls without prior GD. The studies differed in population size, geographic setting, length of follow-up, and methods used to assess NAFLD (CT, ultrasound, FibroScan CAP, or validated indices).

### 3.2. Primary Analysis

**Figure 1:**
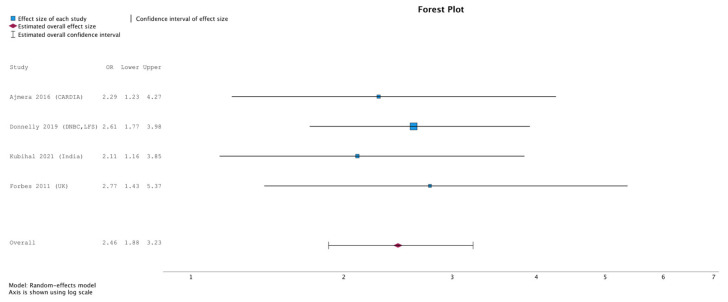
Forest plot of pooled odds ratios for NAFLD in women with prior GD. A random-effects meta-analysis using the REML method showed that women with prior GD had a significantly higher odds of developing NAFLD compared with controls (pooled OR = 2.46, 95% CI 1.88–3.23, *p* < 0.001). Individual study estimates are shown with 95% confidence intervals, and the pooled estimate is represented by a diamond symbol [19,20,21,22].

In the random-effects model, women with prior GD showed higher odds of NAFLD than controls. The pooled odds ratio was 2.46 (95% CI 1.88–3.23, *p* < 0.001), corresponding to an approximately two-fold increase. Study-specific and pooled estimates are shown in Figure 1.

### 3.3. Heterogeneity

There was no statistically detectable heterogeneity across studies (Q = 0.51, *p* = 0.92; I^2^ = 0%). However, heterogeneity statistics have limited power when few studies are available.

### 3.4. Meta-Regression

**Figure 2:**
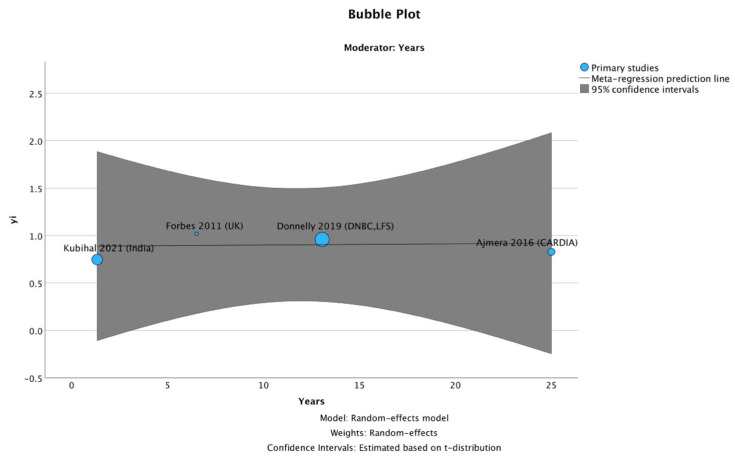
Bubble plot of meta-regression examining follow-up duration as a moderator of the association between GD and NAFLD. A random-effects meta-regression using the REML method with Knapp–Hartung adjustment showed no significant relationship between follow-up duration and effect size (β = 0.001 per year, 95% CI −0.037 to 0.040, *p* = 0.901). Each bubble represents one study, with bubble size proportional to study weight. The fitted regression line and its 95% confidence interval demonstrate that the risk of NAFLD after GD remains stable over time [19,20,21,22].

Meta-regression analysis did not show an association between follow-up duration and effect size (β = 0.001 per year, 95% CI −0.037 to 0.040, *p* = 0.901). This suggests that the magnitude of the association clearly did not vary with time since pregnancy, although the small number of studies limits inference (Figure 2).

### 3.5. Qualitative Assessment of Small-Study Effects

Formal tests for publication bias were not applied because only four studies were included. Instead, the results were assessed qualitatively. An association between prior GD and NAFLD was observed in all included studies, despite differences in study design, population characteristics, follow-up duration, and outcome assessment. Larger cohort studies reported effect estimates similar in size to those from smaller studies, and removal of individual studies did not materially change the pooled estimate. While these observations do not suggest a strong small-study effect, publication bias cannot be excluded. Leave-one-out sensitivity analyses showed that sequential exclusion of individual studies did not materially alter the pooled effect estimate, with detailed results provided in Supplementary Materials Table S3.

## 4. Discussion

Our meta-analysis demonstrates that women with prior GD have a more than two-fold increase of NAFLD, suggesting GD as an early marker of susceptibility to long-term liver metabolic disturbances. Notably, further analysis appears to indicate a potentially permanently increased NAFLD risk over time, extending up to 25 years postpartum. These findings might suggest the importance of long-term follow-up of liver outcomes among women with prior GD beyond the postpartum period. Although the nomenclature of fatty liver disease has recently shifted towards MASLD [23], our analysis is based on available studies that used the traditional NAFLD definition. Consequently, none of the studies required the cardiometabolic risk factor for an MASLD diagnosis, which provides a more comprehensive pathophysiological framework.

Similarly, retrospective studies by Cho et al. (2023) and Lavrentaki et al. (2019) showed a higher prevalence of NAFLD among women with prior GD, even after adjusting for potential confounders (age, BMI, and postpartum metabolic status) [24,25]. Also, the previously published meta-analysis by Foo et al. 2024 reported a 50% higher risk of NAFLD among women with previous GD, evaluating follow-up duration in a subgroup analysis [26]. In favor of these results of individual studies as well as meta-analysis, our meta-analysis quantitatively synthesizes data across populations at risk and different follow-up periods. Furthermore, our results suggest that the increased risk of NAFLD following GD does not attenuate or escalate over time, supporting the interpretation of a sustained and time-independent association.

At the molecular level, the link between GD and subsequent NAFLD reflects a complex association which understanding is crucial to explain the risk that persists even decades after delivery. Moreover, the link between GD and NAFLD has been studied in both directions. The association between GD and NAFLD particularly involves insulin resistance, inflammation, and altered lipid metabolism [8,9,10,11,12]. Pregnancy itself represents a physiological insulin resistance, which is normally compensated for by insulin secretion. In women developing GD, this compensation is inadequate, unmasking pre-existing insulin resistance and predisposing women to persistent metabolic dysfunction in the postpartum period [27,28]. Women with prior GD have decreased insulin sensitivity and compensatory hyperinsulinemia, which may stimulate hepatic lipogenesis. Although insulin resistance seemed to be the key player in developing NAFLD through mediation analysis, it has been demonstrated that it explained less than 10% of the association between prior GD and incident NAFLD [24]. Also, the systemic inflammation among women with GD is further aggravated by placental pro-inflammatory cytokine production, which might additionally promote de novo lipogenesis [29,30]. Shared molecular mechanisms, such as increased hepatic de novo lipogenesis, impaired mitochondrial ß-oxidation, and altered secretion of adipokines, likely contribute to the persistent liver metabolic dysfunction [8,9,10,11,12]. However, a notable limitation of our meta-analysis is the inability to assess the contribution of subsequent glycemic impairment, namely the development of prediabetes or T2D, in mediating the risk of NAFLD. Although glycemic disturbances significantly increase the risk of NAFLD, the included studies did not consistently report glycemic status or de novo diagnosis of T2D during follow-up. As a result, we could not evaluate whether the observed association of prior GD and NAFLD is independent of postpartum glycemic status or is partly mediated by it. Additionally, another major limitation of the analyzed studies and consequently our meta-analysis is the absence of any baseline NAFLD screening, which may attenuate the temporal relationship by the possibility that NAFLD was present before or at any time during the index pregnancy. Furthermore, heterogeneity in NAFLD diagnostic methods should be acknowledged. Such methodological heterogeneity may have influenced the estimated associations with outcomes in our meta-analysis, as less sensitive methods could underestimate risk by misclassifying mild disease, while more precise imaging-based methods likely provide more accurate effect estimates. In that context, methodological harmonization of NAFLD diagnosis across powered studies would improve the comparability of future evidence.

In GD, chronic insulin resistance promotes hepatic de novo lipogenesis through the upregulation of sterol regulatory element-binding protein 1c (SREBP-1c) and carbohydrate-responsive element-binding protein (ChREBP), enhancing triglyceride synthesis while suppressing β-oxidation via reduced peroxisome proliferator-activated receptor-α (PPAR-α) activity [30,31]. The resulting lipid accumulation is further aggravated by increased circulating free fatty acids and impaired very-low-density lipoprotein (VLDL) secretion.

In addition, systemic and placental inflammation in GD leads to elevated TNF-α and IL-6, which activate NF-κB and c-Jun N-terminal kinase (JNK) signaling, promoting hepatocellular inflammation and apoptosis [32,33]. These cytokines also impair insulin receptor substrate (IRS) phosphorylation, further enhancing insulin resistance. Moreover, hyperglycemia-induced oxidative stress generates excessive reactive oxygen species (ROS), which damage mitochondrial DNA and proteins, impairing β-oxidation and perpetuating lipid peroxidation [34].

Altered adipokine secretion represents another mechanism: reduced adiponectin levels diminish AMPK activation, leading to enhanced lipogenesis, while increased leptin promotes profibrogenic TGF-β and STAT3 signaling [35,36]. Furthermore, epigenetic modifications, such as DNA methylation of PPARγ and SREBF1, have been identified in women with prior GD, suggesting a long-term transcriptional “memory” that predisposes to persistent hepatic lipid accumulation [36,37].

Recent evidence highlights the gut–liver axis as a key molecular link between GD and the later development of NAFLD. Dysbiosis observed in GD, characterized by reduced microbial diversity, enrichment of pro-inflammatory taxa, and altered short-chain fatty acid production, can persist postpartum and influence hepatic lipid metabolism and insulin signaling [37,38]. Microbiota-derived metabolites, such as bile acids, endotoxins, and SCFAs, regulate hepatic inflammation, lipogenesis, and fibrosis through the FXR, TGR5, and Toll-like receptor (TLR) pathways, thereby promoting hepatic steatosis. Low-grade endotoxemia caused by increased intestinal permeability further contributes to mitochondrial stress and immune activation in the liver. These mechanisms may explain the interindividual variability in metabolic recovery after pregnancy and suggest that gut-microbiota signatures could serve as biomarkers for stratifying NAFLD risk in women with prior GD. Targeted modulation of the gut–liver axis, through dietary interventions, probiotics, bile acid receptor agonists, or microbiota-directed therapies, represents a promising preventive and therapeutic approach within precision hepato-metabolic medicine.

Furthermore, mitochondrial dysfunction is increasingly recognized as a pivotal mechanism linking GD to long-term hepatic metabolic impairment. Chronic hyperglycemia and elevated free fatty acid flux during GD enhance mitochondrial workload and promote excessive ROS generation, leading to oxidative damage of mitochondrial membranes, proteins, and mtDNA [39,40]. These changes impair β-oxidation and disrupt the electron transport chain, resulting in incomplete fatty acid oxidation and lipid accumulation in hepatocytes. The imbalance between ROS production and antioxidant defenses activates stress-related pathways such as JNK and NF-κB, further amplifying inflammation and insulin resistance. Furthermore, impaired mitochondrial signaling may also attenuate responsiveness to GLP-1-mediated metabolic benefits, suggesting that mitochondrial dysfunction represents not only a consequence but also a potential amplifier of hepatic insulin resistance in women with prior GD [39,40].

These molecular abnormalities create a self-perpetuating cycle of oxidative stress, lipotoxicity, and metabolic inflexibility that predisposes to NAFLD progression.

Taken together, these mechanisms support the concept that GD induces a long-lasting metabolic and molecular imprint characterized by hepatic lipid dysregulation, mitochondrial dysfunction, and inflammatory activation, which together create a sustained vulnerability to NAFLD many years after delivery.

Previously, it was shown that glucagon-like peptide 1 (GLP-1) might be recognized as a key regulator of both glucose homeostasis and liver metabolism [39]. On the other hand, the gap in evidence also limits the ability to explore the involvement of specific hormonal or molecular pathways that may mediate the transition from GD to long-term hepatic metabolic dysfunction [41,42]. Beyond its well-known role in glucose homeostasis, glucagon-like peptide-1 (GLP-1) exerts multiple direct and indirect effects on hepatic metabolism. Activation of the GLP-1 receptor (GLP-1R), a G-protein-coupled receptor expressed in hepatocytes, pancreatic β-cells, and endothelial cells, triggers adenylate cyclase activation and increased intracellular cAMP levels, which subsequently stimulate protein kinase A (PKA) and AMP-activated protein kinase (AMPK) signaling [43,44]. These pathways suppress SREBP-1c-mediated de novo lipogenesis and promote mitochondrial β-oxidation, thereby reducing hepatic triglyceride accumulation [45]. In addition, GLP-1R signaling exerts potent anti-inflammatory and antioxidant effects via inhibition of NF-κB and JNK activation and upregulation of Nrf2, protecting hepatocytes from oxidative stress and apoptosis [46].

In women with prior GD, impaired GLP-1 secretion or reduced GLP-1R responsiveness has been demonstrated postpartum, leading to decreased insulinotropic and lipolytic responses [42]. These defects may perpetuate hepatic insulin resistance and promote lipid accumulation independently of glycemic status. Moreover, emerging data suggest that reduced incretin activity contributes to dysregulation of hepatic autophagy and mitochondrial function, key features of early NAFLD [47]. Collectively, these findings indicate that GLP-1 pathway dysfunction represents a plausible molecular link between GD and future NAFLD and highlights incretin signaling as a potential therapeutic target for metabolic liver disease prevention in this population.

Furthermore, recent work suggests that women with GD may express metabolically heterogeneous patterns, namely a predominant defect in insulin sensitivity or secretion, or mixed [48]. Although these subtypes differ, each of them is followed by an increased risk of developing postpartum glycemic disorder [49]. Since insulin resistance is significant in NAFLD, it is questionable whether subtypes differ with respect to NAFLD risk.

Beyond clinical predictors, molecular stratification may further refine NAFLD risk assessment among women with prior GD. Distinct molecular signatures, including profiles of circulating lipids, adipokines, inflammatory mediators, and gut-microbiota-derived metabolites, reflect heterogeneous metabolic adaptations after pregnancy. Integrative omics approaches (metabolomics, transcriptomics, microbiome profiling) have begun to identify endotypes characterized by differential activation of hepatic lipogenesis, mitochondrial dysfunction, immune/inflammatory signaling, and gut–liver axis perturbation [50,51]. Such molecular phenotyping may help distinguish transient gestational metabolic stress from persistent pathophysiological remodeling predisposing to NAFLD. For example, multi-biomarker panels involving TNF, IL6, SPP1, and CCL5 have been proposed for risk stratification and subtype classification in NAFLD [38]. Moreover, understanding these mechanisms opens perspectives for targeted interventions: modulation of the gut–liver axis (probiotics, bile acid receptor agonists), use of insulin sensitizers or incretin-based agents, as well as personalized nutrigenomic and microbiota-directed therapies could be tailored to individual molecular profiles [39,52,53].

Future research and clinical practice should consider screening for NAFLD among women with prior GD, particularly since the coexistence of these conditions further amplifies cardiovascular risk. Also, large cohorts with longitudinal follow-up focusing on metabolic phenotyping with respect to future NAFLD risk are needed to identify which GD subtype is most likely to develop NAFLD. Moreover, therapeutic strategies targeting the dominant pathophysiological mechanisms, such as insulin sensitizers, incretin-based agents, or weight-loss strategies, could be tailored to individual metabolic profiles.

The inclusion of longitudinal data strengthens the evidence for a sustained and persistent risk across different populations and study designs. Nevertheless, some limitations should be acknowledged. First of all, PubMed was used as the primary database, considering it sufficient to capture all relevant studies on the topic. We were unable to evaluate the effect of postpartum glycemic disorder (prediabetes or diabetes) on the development of NAFLD. The bidirectional link between GD and NAFLD also suggests the possibility that some of the women might have had undiagnosed NAFLD before pregnancy, since baseline liver assessment was not performed. Also, across the included studies, NAFLD was diagnosed using different diagnostic tools. Furthermore, due to the lack of reporting data in the original studies, some factors (e.g., ethnicity) that influence the risk of NAFLD could not be analyzed.

## 5. Conclusions

In conclusion, the results of our meta-analysis show that a history of GD might be associated with a higher risk of NAFLD in later life. Recognizing this connection offers an opportunity to expand postpartum follow-up strategies beyond glycemic outcomes and to intervene earlier in the metabolic–hepatic disease continuum. Integration of liver steatosis screening in follow-up protocols for women with prior GD may contribute to the prevention of hepatic and metabolic complications. Given the shared molecular mechanisms between GD and NAFLD, such as persistent insulin resistance, low-grade inflammation, and dysregulation of the gut–liver axis, these women may represent a unique at-risk phenotype that warrants personalized surveillance and management. Future studies integrating molecular and metabolic phenotyping, including lipidomic, transcriptomic, and microbiome-based biomarkers, could enable stratification of NAFLD risk and facilitate precision prevention strategies. Furthermore, therapeutic interventions targeting key pathophysiological pathways, such as insulin sensitizers, incretin-based therapies, and microbiota-modulating approaches, hold promise for mitigating long-term hepatic and cardiometabolic sequelae in this population.

## Supplementary Materials

The following supporting information can be downloaded at: https://www.mdpi.com/article/10.3390/jcm15031209/s1, PRISMA 2020 Checklist; Table S1: Scoring scheme follows NOS; Table S2: Excluded full-text studies with reasons; Table S3: Leave-one-out sensitivity analysis. Figure S1: PRISMA 2020 flow diagram of study selection.

## Abbreviations

The following abbreviations are used in this manuscript:

GD, GDM	Gestational diabetes
NGT	Normal glucose tolerance
NAFLD	Non-alcoholic fatty liver disease
MASLD	Metabolic dysfunction-associated steatotic liver disease
MASH	Metabolic dysfunction-associated steatohepatitis
T2D	Type 2 diabetes
IR	Insulin resistance
BMI	Body mass index
FLI	Fatty liver index
HSI	Hepatic steatosis index
LFS	Liver fat score
MRI	Magnetic resonance imaging
MR-based	Magnetic resonance-based
MRI-PDFF	Magnetic resonance imaging proton density fat fraction
CT	Computed tomography
CAP	Controlled attenuation parameter
OGTT	Oral glucose tolerance test
WHO	World Health Organization
IADPSG	International Association of Diabetes and Pregnancy Study Groups
NOS	Newcastle–Ottawa Scale
GLP-1	Glucagon-like peptide-1
GLP-1R	Glucagon-like peptide-1 receptor
PRISMA	Preferred Reporting Items for Systematic Reviews and Meta-Analyses
PROSPERO	International Prospective Register of Systematic Reviews

## Appendix A

**Table A1:** Study characteristics.

First Author	Year	Country	Study Design	GDM (n)	NGT (n)	GDM Diagnosis Criteria	NAFLD Diagnosis Method	Follow-Up Duration	Main Findings
Ajmera	2016	USA	Prospective cohort, 25-years follow-up	124	991	Self-report validated by glucose testing	CT scan, liver attenuation ≤40 HU	25 years	OR 2.29 (95% CI 1.23–4.27) for NAFLD with prior GDM
Donnelly	2019	Denmark	Prospective cohort, 9–16 years postpartum	607	619	Danish registry + medical records (validated, 96% sensitivity)	Liver fat indices (LFS, FLI, HSI)	9–16 years	RR 2.34 (95% CI 1.68–3.27) for elevated LFS
Forbes	2011	UK	Cross-sectional recall, 1–9 years postpartum	110	113	WHO 2 h 75 g OGTT criteria	Ultrasound	1–9 years	NAFLD prevalence 38% vs. 17%; OR 2.77 (95% CI 1.43–5.37)
Kubihal	2021	India	Cross-sectional, clinic based, 2018–2019	201	108	IADPSG criteria (FPG ≥ 5.1, 1 h ≥ 10.0, 2 h ≥ 8.5)	Ultrasound + Fibroscan (CAP ≥ 270 dB/m)	Median 16 months	NAFLD prevalence 62.7% vs. 50%; OR 2.11 (95% CI 1.16–3.85)
