# Supplementary material for: Gestational Diabetes Associated with Postpartum NAFLD Risk Meta-Analysis: Evidence for Sustained Metabolic Dysfunction Beyond Pregnancy
Source: J Clin Med. 2026 Feb 4;15(3):1209. doi: 10.3390/jcm15031209 (PMC12898346; doi:10.3390/jcm15031209)
Supplement: Supplementary file 1 [file jcm-15-01209-s001.zip › jcm-4003530-supplementary.pdf]

Supplementary materials

The PubMed search string:

("Diabetes, Gestational"[Mesh] OR "gestational diabetes"[tiab] OR GDM[tiab])

AND

("Non-alcoholic Fatty Liver Disease"[Mesh] OR NAFLD[tiab] OR MASLD[tiab]

OR "hepatic steatosis"[tiab] OR "fatty liver"[tiab] OR "Fatty Liver"[Mesh]).

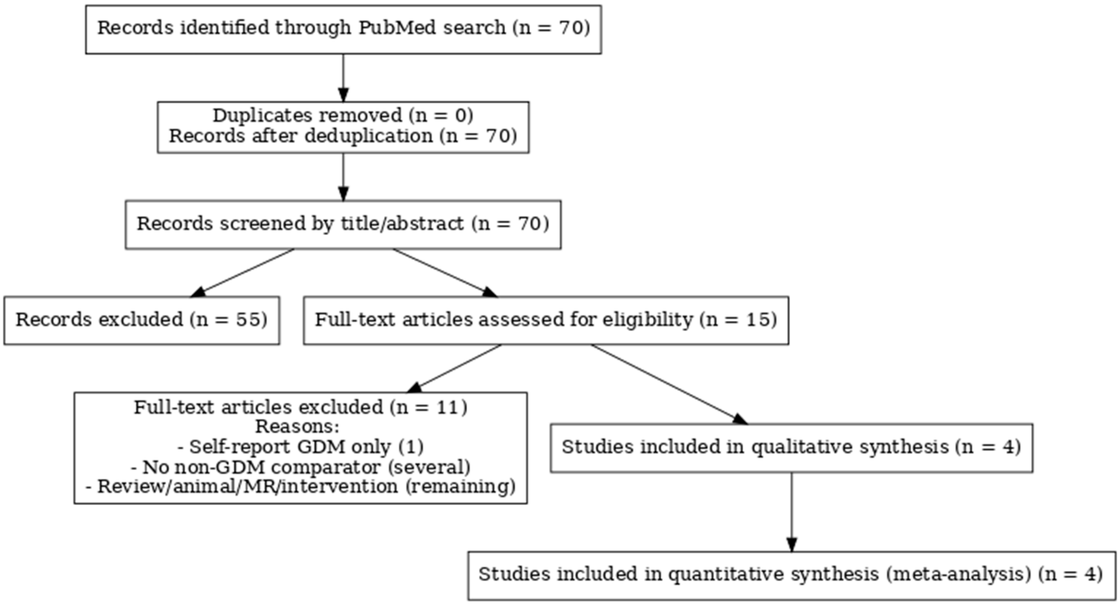

Figure S1: PRISMA 2020 flow diagram of study selection for the meta-analysis of GD history and risk of NAFLD in later life.

A total of 70 records were identified through PubMed. After screening titles and abstracts, 55 were excluded. Fifteen full-text articles were assessed for eligibility, of which 11 were excluded. Four studies met inclusion criteria and were included in the quantitative synthesis.

Newcastle–Ottawa Scale (NOS) – Risk of Bias Assessment:

Table S1. Scoring scheme follows NOS (maximum 9 points). For cohort studies: Selection (0–4), Comparability (0–2), Outcome (0–3). For case–control/cross-sectional designs: Selection (0–4), Comparability (0–2), Exposure/Outcome (0–3).

Study (Design)	Selection (0–4)	Comparability (0–2)	Outcome/Exposure (0–3)	Total (0–9)	Quality
Ajmera et al., 2016 (cohort)	4	2	3	9	High

Donnelly et al., 2019 (cohort)	3	1	2	6	Moderate
Forbes et al., 2011 (case–control)	3	2	2	7	High
Kubihal et al., 2021 (case–control)	3	2	3	8	High

Justifications (abbreviated)

Ajmera 2016: Community-based cohort (CARDIA) with population sampling and long follow-up; GDM exposure validated against records in a subsample (100% sensitivity, 92% specificity); NAFLD assessed by CT at Year 25; follow-up adequate (72% at Year 25).

Donnelly 2019: Population-based cohort (DNBC); GDM status from registry/interviews validated against medical records (~96 % sensitivity); NAFLD estimated by validated indices (LFS, FLI, HSI); 9–16 years follow-up; participation <80 % and limited baseline hepatic data → Outcome 2/3, Comparability 1/2.

Forbes 2011: Retrospective recruitment from antenatal databases; controls drawn from the same source; NAFLD assessed by blinded ultrasound; adequate comparability and outcome assessment; overall high-quality study.

Kubihal 2021: Case–control design (prior vs no GDM); NAFLD assessed by ultrasound and FibroScan CAP with blinded operators; multivariable adjustment for key metabolic and postpartum confounders; high-quality outcome ascertainment.

Study-level Data Extraction Sheet

Table S2. Excluded full-text studies with reasons.

Study	Reason for exclusion
Cho et al., 2023	Exposure = self-reported GDM only, not validated by medical records → risk of misclassification
Zhang et al., 2022 (lipid dysregulation review)	Narrative review, no original NAFLD outcome data

Sethasine & Phaloprakarn, 2021 (breastfeeding)	Exposure = breastfeeding, all women had prior GDM; no non-GDM comparator
Sethasine et al., 2022 (contraception)	Exposure = contraceptive method within prior GDM group; no non-GDM comparator
Hussain et al., 2021	Postpartum NAFLD assessment by FibroScan; no stratification by prior GDM vs. non-GDM
Foo et al., 2024	Systematic review/meta-analysis, not primary data
Lavrentaki et al., 2019	Systematic review/meta-analysis with UK cohort, but outside prespecified inclusion (overlap with included studies)
Animal/murine models (Hribar 2024, Shryack 2025, etc.)	Not human studies
Intervention studies (e.g., liraglutide in prior GDM women)	Exposure = pharmacologic treatment, not GDM itself
Other postpartum metabolic studies	No NAFLD outcome measured
Other liver disease studies (AFLP, alcoholic, viral hepatitis)	Wrong outcome; not NAFLD/MASLD

Leave-one-out sensitivity analysis

Table S3: Leave-one-out sensitivity analysis performed to assess the robustness of the pooled association between prior gestational diabetes and NAFLD, using a random-effects model with REML estimation. *τ^2 values are shown as approximate because the between-study variance was close to zero.

Excluded study	Pooled OR	95% CI (OR)	Log (OR)	SE	Z	p	I^2	τ^2
Ajmera 2016	2.51	(1.86, 3.38)	0.919	0.153	6.013	<0.0001	0%	0.000
Donnelly 2019	2.35	(1.64, 3.38)	0.856	0.185	4.637	<0.0001	0%	0.000
Kubihal 2021	2.56	(1.89, 3.47)	0.941	0.154	6.105	<0.0001	0%	0.000
Forbes 2011	2.41	(1.79, 3.23)	0.878	0.151	5.826	<0.0001	0%	0.000
