# Supplementary material for: Gestational Diabetes Associated with Postpartum NAFLD Risk Meta-Analysis: Evidence for Sustained Metabolic Dysfunction Beyond Pregnancy
Source: J Clin Med. 2026 Feb 4;15(3):1209. doi: 10.3390/jcm15031209 (PMC12898346; doi:10.3390/jcm15031209)
Supplement: Supplementary file 1 [file jcm-15-01209-s001.zip › PRISMA_2020_checklist (1)24.01.2026.pdf]

PRISMA 2020 Checklist

Section and Topic	Item #	Checklist item	Location where item is reported
**TITLE**			
Title	1	Identify the report as a systematic review.	Title, Abstract
**ABSTRACT**			
Abstract	2	See the PRISMA 2020 for Abstracts checklist.	Abstract(Background/Objectives, Methods, Results, Conclusions)
**INTRODUCTION**			
Rationale	3	Describe the rationale for the review in the context of existing knowledge.	Introduction (Paragraphs 1-3)
Objectives	4	Provide an explicit statement of the objective(s) or question(s) the review addresses.	Introduction (Final paragraph)
**METHODS**			
Eligibility criteria	5	Specify the inclusion and exclusion criteria for the review and how studies were grouped for the syntheses.	Section 2.1 Eligibility Criteria
Information sources	6	Specify all databases, registers, websites, organisations, reference lists and other sources searched or consulted to identify studies. Specify the date when each source was last searched or consulted.	Section 2.2 Information sources and search strategy (PubMed from inception do 30th June 2025; reference list screening)
Search strategy	7	Present the full search strategies for all databases, registers and websites, including any filters and limits used.	Appendix A
Selection process	8	Specify the methods used to decide whether a study met the inclusion criteria of the review, including how many reviewers screened each record and each report retrieved, whether they worked independently, and if applicable, details of automation tools used in the process.	Section 2.3. Study selection (four reviewers independently screened; discrepancies resolved by discussion)
Data collection process	9	Specify the methods used to collect data from reports, including how many reviewers collected data from each report, whether they worked independently, any processes for obtaining or confirming data from study investigators, and if applicable, details of automation tools used in the process.	Section 2.5 Data extraction and risk of bias (two reviewers independently extracted data into standardized template)
Data items	10a	List and define all outcomes for which data were sought. Specify whether all results that were compatible with each outcome domain in each study were sought (e.g. for all measures, time points, analyses), and if not, the methods used to decide which results to collect.	Section 2.1 Eligibility Criteria (NAFLD/MASLD diagnosed by imaging or validated index); Section 2.5
	10b	List and define all other variables for which data were sought (e.g. participant and intervention characteristics, funding sources). Describe any assumptions made about any missing or unclear information.	Section 2.5 Data extraction and risk of bias (study design, sample size, population characteristics, exposure and outcome ascertainment, effect estimates)
Study risk of bias assessment	11	Specify the methods used to assess risk of bias in the included studies, including details of the tool(s) used, how many reviewers assessed each study and whether they worked independently, and if applicable, details of automation tools used in the process.	Section 2.5 Data extraction and risk of bias (New Castle -Ottawa Scale; study scoring ≥ 7 considered high quality)
Effect measures	12	Specify for each outcome the effect measure(s) (e.g. risk ratio, mean difference) used in the synthesis or presentation of results.	Section 2.6 Effect measures and synthesis (log odds ratios; ODs ratios with 95% CI)
Synthesis methods	13a	Describe the processes used to decide which studies were eligible for each synthesis (e.g. tabulating the study intervention characteristics and comparing against the planned groups for each synthesis (item #5)).	Section 2.1 Eligibility Criteria; Section 2.3 Study Selection
	13b	Describe any methods required to prepare the data for presentation or synthesis, such as handling of missing summary statistics, or data conversions.	Section 2.6 Effect Measures and Synthesis (effect sizes transformed to log odds ratios)
	13c	Describe any methods used to tabulate or visually display results of individual studies and syntheses.	Section 2.6 (Forest plot; Appendix A)

PRISMA

PRISMA 2020 Checklist

Section and Topic	Item #	Checklist item	Location where item is reported
	13d	Describe any methods used to synthesize results and provide a rationale for the choice(s). If meta-analysis was performed, describe the model(s), method(s) to identify the presence and extent of statistical heterogeneity, and software package(s) used.	Section 2.6 Effect measures and synthesis (Random effects Model with REML; Cochran Q, τ^2, I^2; SPSS statistics 31.0)
	13e	Describe any methods used to explore possible causes of heterogeneity among study results (e.g. subgroup analysis, meta-regression).	Section 2.6 Effect measures and synthesis (random effects meta-regression for follow up duration; Knapp Hartung adjustment)
	13f	Describe any sensitivity analyses conducted to assess robustness of the synthesized results.	Section 2.6 Effect measures and synthesis (leave one out sensitivity analysis)
Reporting bias assessment	14	Describe any methods used to assess risk of bias due to missing results in a synthesis (arising from reporting biases).	Section 2.7 Additional analysis (formal test not applied due to N=4; qualitative assessment of small study effects)
Certainty assessment	15	Describe any methods used to assess certainty (or confidence) in the body of evidence for an outcome.	Not formally applied
**RESULTS**			
Study selection	16a	Describe the results of the search and selection process, from the number of records identified in the search to the number of studies included in the review, ideally using a flow diagram.	Section 2.3 Study selection (70 records; 55 excluded at screening; 15 full texts assessed; 4 included);Supplementary Materials Figure S1
	16b	Cite studies that might appear to meet the inclusion criteria, but which were excluded, and explain why they were excluded.	Appendix A (Excluded full text studies with reasons)
Study characteristics	17	Cite each included study and present its characteristics.	Section 2.4 Included studies and GD diagnostic criteria(Ajmera 2016, Donnelly 2019, Forbes 2011, Kubihal 2021); Appendix A
Risk of bias in studies	18	Present assessments of risk of bias for each included study.	Supplementary Materials (risk of bias table)
Results of individual studies	19	For all outcomes, present, for each study: (a) summary statistics for each group (where appropriate) and (b) an effect estimate and its precision (e.g. confidence/credible interval), ideally using structured tables or plots.	Results Section (forest plot); Appendix A (Data extraction sheet, Table A_1)
Results of syntheses	20a	For each synthesis, briefly summarise the characteristics and risk of bias among contributing studies.	Section 2.4; Section 2.5; Results
	20b	Present results of all statistical syntheses conducted. If meta-analysis was done, present for each the summary estimate and its precision (e.g. confidence/credible interval) and measures of statistical heterogeneity. If comparing groups, describe the direction of the effect.	Abstract/Results(OR 2.46, 95% CI 1.88-3.23, p<0.001
	20c	Present results of all investigations of possible causes of heterogeneity among study results.	Abstract/Results(meta-regression: β =0.001 per year, 95% CI -0.037, 0.040, p=0.901)
	20d	Present results of all sensitivity analyses conducted to assess the robustness of the synthesized results.	Appendix A (leave one out analysis)
Reporting biases	21	Present assessments of risk of bias due to missing results (arising from reporting biases) for each synthesis assessed.	Section 2.7, Additional analysis(qualitative assessment: formal test not reliable when N=4)

PRISMA 2020 Checklist

Section and Topic	Item #	Checklist item	Location where item is reported
Certainty of evidence	22	Present assessments of certainty (or confidence) in the body of evidence for each outcome assessed.	Not formally applied
**DISCUSSION**			
Discussion	23a	Provide a general interpretation of the results in the context of other evidence.	Discussion
	23b	Discuss any limitations of the evidence included in the review.	Discussion
	23c	Discuss any limitations of the review processes used.	Discussion
	23d	Discuss implications of the results for practice, policy, and future research.	Abstract/Conclusions; Discussion
**OTHER INFORMATION**			
Registration and protocol	24a	Provide registration information for the review, including register name and registration number, or state that the review was not registered.	Section 2. Materials and Methods ('protocol was not prospectively registered)
	24b	Indicate where the review protocol can be accessed, or state that a protocol was not prepared.	Section 2. Materials and Methods ('protocol was not prospectively registered)
	24c	Describe and explain any amendments to information provided at registration or in the protocol.	N/A (no registration)
Support	25	Describe sources of financial or non-financial support for the review, and the role of the funders or sponsors in the review.	Funding
Competing interests	26	Declare any competing interests of review authors.	Conflicts of interest
Availability of data, code and other materials	27	Report which of the following are publicly available and where they can be found: template data collection forms; data extracted from included studies; data used for all analyses; analytic code; any other materials used in the review.	Section 2.9, Supplementary Materials; Appendix A

From: Page MJ, McKenzie JE, Bossuyt PM, Boutron I, Hoffmann TC, Mulrow CD, et al. The PRISMA 2020 statement: an updated guideline for reporting systematic reviews. BMJ 2021;372:n71. doi: 10.1136/bmj.n71.
This work is licensed under CC BY 4.0. To view a copy of this license, visit https://creativecommons.org/licenses/by/4.0/
